# Ghostbuster—A Western Blot-Based Panel Method to Resolve False-Positive Brucellosis Serology Test Results

**DOI:** 10.3390/microorganisms13030574

**Published:** 2025-03-03

**Authors:** Borbála Bányász, József Antal, Béla Dénes

**Affiliations:** 1 Institute of Isotopes Co., Ltd., 1121 Budapest, Hungary; banyasz@izotop.hu; 2 Omixon Biocomputing Ltd., 1117 Budapest, Hungary; 3 Department of Microbiology and Infectious Diseases, University of Veterinary Medicine Budapest, 1143 Budapest, Hungary; 4 National Laboratory of Infectious Animal Diseases, Antimicrobial Resistance, Veterinary Public Health and Food Chain Safety, University of Veterinary Medicine Budapest, 1078 Budapest, Hungary

**Keywords:** brucellosis, serology, false-positive serologic results, protein antigen panel, western blotting

## Abstract

False-positive serologic results (FPSRs) of brucellosis occur from time to time in various livestock with all the consequences (quarantine, compulsory slaughter, etc.) that follow true-positive laboratory results. A method based on the Polyacrylamide Gel Electrophoresis/Western Blot of a protein panel for resolving the FPSRs in the diagnosis of brucellosis was developed. Within the context of limited positive serum sample availability in Europe, the method successfully discriminates *Brucella*-positive sera from samples containing antibodies raised against infections caused by other Gram-negative bacteria causing FPSRs. An average CV% of 1.36 was determined for both repeatability and reproducibility for the whole separation mw range, and the test achieved 1.00 Diagnostic Sensitivity and 1.00 Diagnostic Specificity. The method with pre-prepared WB panels provides a rapid (less than 3 h), easily standardizable, and validatable alternative to existing confirmation methods. The whole WB process of the *Brucella* proteins and the subsequent densitometry can be accomplished with commercially available equipment, ready-to-use reagents, and open-source software, providing cost-effectiveness. The results of this study could attract broader attention, since molecular species in the 35.0–75.0 kDa range can serve as antigens in *Brucella* serology and the same fraction can be considered in the development of synthetic *Brucella* vaccines.

## 1. Introduction

From the last decades of the 20th century onward, European Union member states have been considered bovine brucellosis-free territories. The human health consequences of this zoonosis practically disappeared with the radical eradication of *Brucella abortus*, the primary agent of bovine infections. However, the brucellosis problem remains with us. One could say ‘A spectre is haunting Europe—the spectre of brucellosis’. False-positive serologic results—the ghosts of brucellosis—occur from time to time in various livestock with all the grim consequences (quarantine, compulsory slaughter, etc.) that follow true-positive laboratory results.

The pathogens behind brucellosis are the *Brucella* species: there are more than 30 members of this genus, including isolates from exotic hosts like cetaceans or the surface of a human breast implant [1] and the recently isolated *Brucella nosferati* from tropical vampire bats, which forage on both humans and animals, increasing the potential for zoonotic transmission [2]. These findings demonstrate the physiological and genetic flexibility of the bacteria. The *Brucella* genus is characterized by a wide host variety, environmental persistence (against extreme temperature, pH, and humidity) outside the host [3], the ability of intracellular localization within the host organism [4,5,6], and the mild toxicity of the *Brucella* endotoxin [7]—three orders of magnitude lower compared to the corresponding *E. coli* endotoxin. Each one of these features alone could place the recognition of brucellosis in twilight. However, together, they have produced a diagnostic nightmare, present since the isolation of the pathogen by Bruce and the detection of *Brucella* infection by Wright using the first serologic diagnostic probe at the end of the 19th century [8,9,10].

The most widely applied methods to diagnose brucellosis are serologic tests, among them being a modified variety of the very first one mentioned above [11,12]. Historically, Rose Bengal test (RBT) [13] and serum agglutination test (SAT) [3,14,15,16] were used. Then, the robust but cumbersome complement fixation test (CFT) [17,18] was adapted, which became the confirmatory test of brucellosis recommended and defined by the World Organization for Animal Health (WOAH, formerly the Office International des Epizooties—OIE). For decades, laboratories have also used brucellae-specific Enzyme-Linked Immunosorbent Assay (ELISA) tests. However, ELISA remains a screening test only, necessitating confirmation of its results. This is mostly the case for positive results, since, given the binary classification diagnostic parameters of *Brucella* iELISA tests, if false-positive serologic results (FPSRs) are considered, high sensitivity and poor specificity will be experienced, usually at >97% and <60%, respectively, with 95% confidence [19].

Currently, the application of WOAH-recommended serologic tests for screening serum samples and/or the confirmation of serologic results is a mandatory requirement by Hungarian law (12/2008. (II. 14) FVM), and state-of-the-art or even advanced nucleic acid-based tests (quantitative PCR, cell-free DNA–Next-Generation Sequencing) are only recommended for the identification of the pathogen.

The risk of false positivity in the widely used serologic ELISA tests for the detection of brucellosis is so high that some laboratories not only use the CFT but also run the classic RBT and SAT in parallel with it in order to mitigate the problem.

Despite the use of multiple confirmatory tests, false-positive results can occur among the results of confirmatory tests as well—thereby reinforcing them as real positives—as demonstrated below through an unpublished case study carried out by the authors.

### 1.1. The Molecular Background of FP Serologic Test Results

**Figure 1:**
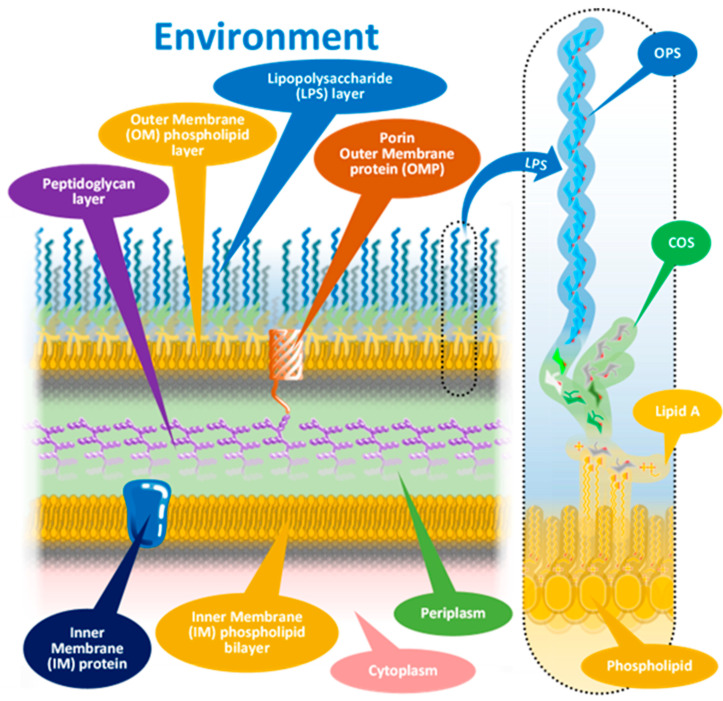
Cell wall structure of Gram-negative bacteria.

The members of *Brucella* spp. as Gram-negative bacteria are characterized by their sandwich-structured cell envelopes composed of the lipopolysaccharide (LPS)-covered bacterial outer membrane and the inner cytoplasmic cell membrane with a thin peptidoglycan layer between them in the periplasmic space, as presented in Figure 1 with an insert of a schematized *Brucella* LPS.

Almost all members of the genus *Brucella* adhere to this schematic; however, with serious biological, biochemical, and subsequently serological consequences, similarly to other Gram-negative bacteria [20], there are some mutant *Brucella* species that lack the vast outer lipopolysaccharide layer [21]. These mutants were identified as R (rough)-type *Brucella* in contrast with the S (smooth)-type species, based on visual characterization of bacterial colonies grown on solid media. The virulence of R-type mutants is radically weakened due to mutations not detailed here [21], making some of them suitable for use as *Brucella* vaccines.

The prevalence of the S-type *Brucella* species is substantially higher, and hence, false positivity in *Brucella* serology is mainly contributed to S types; therefore, the discussion will focus on the biochemical and antigenic character of smooth brucellas.

The outermost part of the bacterium envelope is the cell wall, composed of a phospholipid membrane monolayer and an associated lipopolysaccharide (LPS) layer. The outer membrane also anchors the periplasmic peptidoglycan layer by specific proteins, outer membrane proteins (OMPs). Some OMPs belong to the porin protein family, providing molecular communication across the cell wall. Recent studies [22,23] proved that the LPS is inhomogeneous: a mixture of full-length polysaccharide chains (S LPS) and truncated forms (R LPS) clustered around OMPs.

The long polysaccharide chains of the LPS (built from dozens of monosaccharides) form a protective, fur-like layer, making it difficult for hydrophobic molecules to penetrate the outer membrane and enter the periplasm. This strong fur coat deceives the host immune system during the early phase of the infection and provides protection for *Brucella* cells from monocyte phagocytosis [5,6,7].

As the insert in Figure 1 shows, the LPS consists of three main elements, different in composition, structure, and function. These elements, starting from the interior of the bacterium cell toward the extracellular space, are (i) the O-specific polysaccharide—OPS; (ii) the core oligosaccharide—COS; and (iii) so-called Lipid A.

The Lipid A structures, being the most deeply buried part of the LPS and embedded into the OM, have little importance from a serologic point of view [12].

The core oligosaccharide (COS) is a connecting region between Lipid A and the OPS. The COS’ diverse composition and branched structure could serve a good basis for antigenic differentiation; however, the utilization of this diversity is difficult for serologic applications in the case of S-type bacteria, as most of the COS remains concealed under the OPS layer [12].

The surface part of the LPS, the O-specific polysaccharide (OPS), is composed of a high number of repeating subunits built of sugar components of various compositions. The pattern of subunit repetitions is characteristic of bacterial species, resulting in high antigen variability, which could serve as the basis for the serological grouping of Gram-negative bacteria. The number of sugar moieties (which could be zero in rough mutants) in the O-specific polysaccharide chain determines the morphology of the bacterial colony, that is, smooth or rough colony types with and without lengthy OPS chains, respectively [12].

The O-specific polysaccharide as the outer layer of the cell wall is the main antigen determinant of the LPS and provides the basis for the serologic differentiation of Gram-negative bacteria species and strains [24]. The molecular and topological diversity of the OPS is theoretically high: more than five dozen types of sugar moieties were identified as constituents of the polymer in variable numbers, proportions, and clusters, built into linear or branched chains, linked to even non-sugary substituents [25,26]. Despite this theoretical diversity, the serologic differentiation of Gram-negative bacterial strains is based on the antibody recognition of the repeating oligomeric saccharide motifs in the OPS, rather than on individual sugar moieties [12]. Similarly, more than 180 different O serotypes of *E. coli* were identified before 2005 [26] due to the motif variability. Paradoxically, the high molecular diversity results in lower antigenic diversity in several cases [26]. *E. coli* O35 and *Salmonella enterica* O62, *E. coli* O98 and *Yersinia enterocolitica* O11,24, *E. coli* O8, *Klebsiella pneumoniae* O5, and our current subject, *Brucella* spp., or *E. coli* O157:H7 and *Y. enterocolitica* O9 all have identical or nearly identical antigens, in the latter case identical enough to give false-positive serologic results.

Due to its chemical nature that is abundantly composed of saccharides, and the rather narrow repertoire of sugar moieties occurring across bacterial families, from a serological point of view, the outmost polysaccharide component of the protective LPS layer seems to be overly uniform in the case of smooth Gram-negative bacteria—consequently, the application of isolated antigens with S LPS origins in serological tests almost automatically provides false positives [12].

The cross-reactions resulting from the antigen applied in serologic tests with several Gram-negative bacteria including *Y. enterocolitica* O:9, *E. coli* O:157, *S. urbana* O:30, *E. hermanii*, *Francisella tularensis*, *P. maltophilia* 555, *S. godesberg*, and *Vibrio cholerae* are well documented [19,20,27,28,29,30]. The high prevalence of the above-mentioned and other, still unidentified non-pathogenic bacteria strains that cause mild or asymptomatic disease is a certain and unavoidable condition in our livestock.

### 1.2. Possible Solutions

Since the recognition of the impact of false positivity, several attempts have been made to resolve the problem caused by the application of the S-type LPS as an antigen. These efforts could be classified into three main approaches [12]: ELISA application of (i) non-LPS or non-LPS elements, that is, other components of the bacterium cells, like cytoplasmic (e.g., superoxide dismutase [31]), periplasmic (e.g., protein 26 (BP26) [32]), inner membrane (e.g., ABC transporter [31]), outer membrane (e.g., Omp10, Omp 19, or OMP31, [33] and [34], respectively) origin proteins, or even those of mixed origin like the recombinant fusion protein of Omp16, Omp25, Omp31, Omp2b, and BP26 epitopes [35]; (ii) non-S LPS antigens, that is, either R LPS-based [36] or synthetic oligosaccharide-based tests [27,37,38]. As a third approach, non-ELISA applications should be mentioned, such as the Brucellin-based skin test [39] or the determination of the cell-free DNA of the immune processed bacteria isolated from blood plasma [40,41,42,43].

Although all approaches have provided promising results, a real solution remains elusive, since either the throughput is too low, the costs are too high, early detection is not possible, or there is simply no satisfactory differentiation between *Brucella* and other Gram-negative species. 

We present below our attempt at the resolution of false positives with an approach based on lessons learned from these earlier efforts. The main lessons we considered are the following: (i) protein antigens provide more reliable differentiation due to the superior structural diversity of proteins compared to carbohydrates (sugars), (ii) the application of more different protein antigens (panel test) offers better resolution, which can be enhanced if (iii) the elements of the panel have different cellular origins and (iv) the earlier results could not provide any information about the diversity of the panel (the optimal number and origin of the panel members, their concentration relations (balancing), and the side effects of other targets in terms of detection (multiplexing)); moreover, (v) only sporadic information is available on the effect of parallel paneling of separated protein antigens.

These considerations led us to the application of an (i) antigen panel, consisting of (ii) isolated proteins from (iii) the whole *Brucella abortus* cell, with the panel members separated with the highest possible resolution before serum detection is accomplished. SDS Polyacrylamide Gel Electrophoresis (SDS PAGE), as an easy and cost-effective protein separation method with acceptable resolution in the 10.0 to 250.0 kDA molecular weight (mw) range, in combination with the Western Blotting (WB) immobilization and detection method (horseradish peroxidase conjugated anti bovine antibody–3,3′-Diaminobenzidine (DAB) substrate), followed by optical densitometry, easily satisfies these theoretical features and provides further practical advantages.

The advanced PAGE systems (i) offer ready-made gels with outstanding reproducibility and easy handling and thus can be applied as validatable diagnostic laboratory tools, (ii) their separation runs can be visually followed, and (iii) their costs are negligible compared to other separation means. The blotting system, accompanied by the electrophoretic chambers, also offers high reproducibility and ease of use. The PVDF membranes applied in the Western Blotting procedure as the protein-immobilizing tool provide long-term storage (1–2 months at 4 °C, a half a year at minimum at −20 °C, and in the longer term at −70 °C) [44,45]. In laboratory practice, this means that with a sufficient number of antigen gel separations and generated blots, the same results can be provided through the years without the need for revalidation.

Although not within the scope of this study, an indirect benefit of PAGE/WB application is the easy identification of protein bands: both Edman sequencing and mass spectrometry identification can easily be accomplished by slicing the immobilized protein(s) from the PVDF membrane.

## 2. Materials and Methods

### 2.1. Samples—Case Study

For brucellosis screening, 193 serum samples of gilts (authors’ note: a gilt is a female pig that has not produced a litter of piglets yet or is being grown and finished for slaughter) were received from a population imported for breeding purposes and assumed to be brucellae-free. Further sampling and analysis were performed on a subset of the swine herd (70 specimens for technical/traceability reasons) at two additional time points (Days 41 and 62).

### 2.2. Samples—WB Panel Study

The molecular weight standard (MW STD) applied as an internal control for SDS PAGE separations, retention factor normalization, and mw determination was the Spectra Multicolor Broad Range Protein Ladder provided by Thermo Scientific (Waltham, MA, USA). The MW STD contains 10 proteins with molecular weights of 125.0, 80.0, 70.0, 50.0, 40.0, 30.0, 25.0, 15.0, and 10.0 kDa, stained with 4 colors (blue, orange, green, and pink) as seen in Figure 3A.

As Table 1 shows, altogether, 48 bovine sera were analyzed with origins in 6 different European countries. Two main sample types were used: freshly collected serums received by the laboratory for routine analysis (39 samples, identified by the ear tag of the individual animals) and reference samples from the Animal Health Laboratory of the French Agency for Food Environmental & Occupational Health Safety (ANSES) and from Ukraine, former Soviet Union (9 conserved serums identified as ANSES and CCCP, respectively). According to their ear tags, 26 samples have Hungarian (HU or missing), 9 samples have German (DE), 3 have Danish (DK), and 1 has Dutch (NL) breeding origin.

### 2.3. Serologic Laboratory Tests

ELISA tests were accomplished with the BRUCELISA-M ELISA Kit provided by Diavet Ltd., Budapest, Hungary. The protocol applied was identical to that recommended by the manufacturer.

The complement fixation test, serum agglutination test, and the Rose Bengal test were carried out as laboratory-developed tests (LDTs) based on the protocol recommended by WOAH Chapter 3.1.4.B.2.7 [18]. 

For the CFT, Amboceptor hemolysin and the complement were provided by Institute Virion\Serion GmbH, Würzburg, Germany. Sheep erythrocytes and the Pourquier Veronal Buffer (5x) were purchased from Culex LP, Budapest, Hungary, and from IDEXX, Westbrook, ME, USA, respectively.

The applied antigen in the case of the CFT and SAT was the *Brucella abortus* antigen for SA provided by APHA Scientific, UK, calibrated and adjusted by the manufacturer in order to give a 75% agglutination reaction with a standard positive serum in a dilution of 1:499 in the SAT. The positive serum was the Checking positive *Brucella abortus* serum provided by Bioveta a.s., Ivanovice na Hané, Czech Republic, with a titer of 1000 NE for the prediluted positive serum. For the RBT, Pourquier Rose Bengale Ag provided by IDEXX was used, calibrated and adjusted by the manufacturer to give a positive reaction at a dilution of 1:45 of the second international anti-*Brucella abortus* serum (OIEISS), while, at a dilution of 1:55, the reaction should be negative. The positive and negative controls were provided by VIRCELL S.L, Granada, Spain.

A negative CFT result was determined if 100% of the erythrocytes were hemolyzed. In the case of SAT measurements, agglutination was approved if a pellet could be observed under the clear liquid detected in the wells.

The titer was determined with a five-level positivity scoring (−, + to ++++) system applied for the respective dilution steps. Then, the scores were converted to International CFT Units (ICFTU/mL) with a 20 ICFTU/mL cut-off level and International Units (IU) with a 30 IU cut-off threshold, for CFT and SAT, respectively.

*Yersinia* ELISA tests were carried out with the recomWell *Yersinia* IgG ELISA Kit provided by Mikrogen Diagnostik GmbH, Neuried, Germany, with a modified protocol. The original test antigen was used with the secondary antibody from the BRUCELISA-M ELISA Kit provided by Diavet Ltd. (Budapest, Hungary), and otherwise, the Mikrogen test protocol was followed.

### 2.4. Confirmatory Examinations Other than Serology

In several cases (see Results, Section 3), confirmatory examinations like bacteriology, PCR, or diagnostic autopsy were carried out in external institutions. The methods applied are not detailed here; only the positive or negative results will be shown.

### 2.5. WB Panel Test—Antigen Panel Isolation—Bacterial Culture

*Brucella melitensis* B115, a non-smooth (rough) strain, was used in this study, as recommended by [46], to avoid false-positive reactions caused by the smooth LPS antigen. *B. melitensis* B115 was kindly provided by L’Agence Nationale de Sécurité Sanitaire de L’alimentation, de L’environnement et du travail (ANSES), France. Bacterial cells were maintained as glycerol stocks at −80 °C and the strain was cultured at 37 °C for 48 h under 10% CO_2_ atmosphere in a 2 × 1 L fermentation flask filled with Tryptone Soy broth (provided by Biolab Ltd., Budapest, Hungary) complemented with 5% horse serum. After 48 h, the cell concentration reached approximately 1011 cells/mL density determined by an optical density measurement. The cells were harvested by centrifugation at 4000× *g* for 15 min at 4 °C. The centrifuged pellet of bacterial cells was washed with 0.9% NaCl solution and resuspended in 500 mL 0.9% NaCl solution. The bacteria were killed by the addition of 7.5 mL 1 M peracetic acid to avoid the denaturation and degradation of the proteins. The suspension was incubated overnight at room temperature; then, the killed cells were centrifuged at 9000× *g* for 15 min at 4 °C, and the centrifuged pellet of bacterial cells was resuspended in 50 mL PBS buffer pH 7.2, aliquoted into 1 mL portions, and stored at −20 °C until further use.

### 2.6. WB Panel Test—Antigen Panel Isolation—Protein Isolation

First, 4 × 1 mL of frozen *Brucella melitensis* B115 bacterial culture was thawed at room temperature. The suspension was centrifuged at 4 °C for 15 min with 11,500× *g* force. The supernatants were safely discarded, and the pellets were washed with STE buffer pH 8.0 containing 10 mM TRIS, 100 mM NaCl, and 1 mM EDTA, resuspended, and centrifuged with the same parameters presented above. After the removal of the supernatants, the pellets were suspended in 1–1 mL Trifluoroacetic acid (TFA) and were incubated on ice. After 60 min of incubation, the TFA suspensions were centrifuged as described above. Following two acetone washing–centrifugation cycles, the pellets were dried on a Heto DNA Mini centrifugal evaporator instrument for 2.5 h at 1 mBar. The dry pellets were resuspended in 0.5–0.5 mL 4× diluted Thermo Scientific NuPAGE LDS Sample Buffer and incubated at room temperature overnight. The incubated protein suspension was further homogenized in a vwr Ultrasonic Cleaner for 5 min and then centrifuged at 15 °C for 60 min with 20,000× *g* force. The protein content of the isolate was determined with the Pierce BCA Protein Assay Kit provided by Thermo Scientific (Waltham, MA, USA), resulting in an average 0.8 mg/mL total protein concentration. The isolated protein mixture was aliquoted, and the aliquots were stored at −20 °C until further use.

### 2.7. WB Panel Test—Polyacrylamide Gel Electrophoresis (PAGE)

Polyacrylamide Gel Electrophoresis separations [47] of the isolated *Brucella* proteins were carried out with 12-well NuPAGE 4–12% Bis-Tris Gels assembled into the InvitroGen Mini Gel electrophoresis tank filled with NuPAGE MES-SDS Running Buffer. Then, 20 μL of samples containing 20 μg total protein was applied with 4× diluted NuPAGE LDS Sample Buffer into the gel sample wells. The required concentration was set with the dilution of the isolate with the sample buffer. The 30 min long separating runs were accomplished with 160 mA and a constant 200 V electric current, with power decreasing from 28 W to 16 W. The running conditions were maintained by a PowerEase Touch 300 W power supply. All runs contained at least one lane with protein MW standard Spectra Multicolor Broad Range Protein Ladder. The quality of the separation was checked by staining the gels with either SimplyBlue SafeStain or the Pierce Silver Stain Kit. All equipment and reagents used in PAGE runs were provided by Thermo Scientific, USA, and the protocols were carried out according to the manufacturer’s instructions.

### 2.8. WB Panel Test—Western Blotting—Protein Transfer

Transferring the PAGE-separated protein bands from a gel onto a protein binding PVDF membrane via an electromagnetic field, that is, electroblotting [48], was carried out with the Invitrolon PVDF membrane. The membranes were pre-wetted with 99% methanol for 30 s, rinsed for 30 s with distilled water, sandwiched with blotting pads/blotting filter paper layers, and placed in the Invitrogen Mini Blot Module assembled with the InvitroGen Mini Gel electrophoresis tank filled with 20× diluted Invitrogen Blot Transfer Buffer. The 90 min long transfer runs were accomplished with 230 mA and a constant 20 V electric current, with power decreasing from 4 W to 2 W. The running conditions were maintained by a PowerEase Touch 300 W power supply. All equipment and reagents used for protein transfer were provided by Thermo Scientific, and the protocols were carried out according to the manufacturer’s instructions. The quality of the transfer was checked by visual observation of the appearance of the prestained MW SDT protein bands as shown in Figure 3A. The PVDF membranes were soaked in 99% methanol for 30 s and rinsed for 30 s with distilled water to fix the blotted protein bands. After drying, all the lanes including the MW SDT were individually identified on the membranes, and the membranes were sliced along with the lanes. The membrane slices were wrapped in aluminum foil and stored at −20 °C for further use.

### 2.9. WB Panel Test—Western Blotting—The Development of the Blots

The detection and visualization of the blotted protein bands with the development of the WB panels with serum samples was carried out with an antibody staining colorimetric detection method [49]. The pre-wetted (see above) membrane slices were treated at room temperature for 60 min with Blocker Casein in TBS buffer to block the non-specific binding of the primary antibodies. The blocked membranes were soaked in serum samples containing the primary antibodies. The membranes were removed from the sera and underwent 3 washing cycles consisting of a rinsing step with 20× diluted TBS Tween 20 washing buffer and 10 min incubation at room temperature. The membranes were incubated with the Goat Anti Bovine IgG (H&L) Secondary Antibody, *HRP*, for 60 min at room temperature. After incubation, the membranes underwent 3 washing cycles described above, and the colorimetric detection was carried out with the Membrane Enhanced DAB Substrate Kit for 10 min at room temperature, resulting in well-visualizable brown bands. The antibody conjugated horseradish peroxidase–DAB reaction was stopped by the quick dilution of the reaction mixture with distilled water. All equipment and reagents used for protein transfer were provided by Thermo Scientific, and the protocols were carried out according to the manufacturer’s instructions. The developed membrane slices were dried and stored in a dark place at room temperature for further use and archiving.

### 2.10. WB Panel Test—The Densitometry and Determination of the mw of the Protein Bands on the WB Lanes

The developed blots were scanned with a Canon CanoScan Lide 110 flatbed scanner with 600 dpi resolution. The resulting.jpg images were normalized to a chosen MS standard lane (shown in Figure 3A) via linear transformations. First, the retention range normalization was performed by magnifying or minifying the images of the standard lanes on the individual blots to obtain identical distances between the 10.0 and 235.0 kDa in pixels; then, the 235.0 kDa lanes were matched by a simple linear offset. The magnification and offset values were determined for the standard lanes of each PAGE/WB run and then were applied for the sample lanes. The densitometry of the normalized pictures was accomplished with the GelAnalyzer 23.1 desktop app for 1D gel electrophoresis evaluation software by GelAnalyzer.com. The analytical window of the GelAnalyzer including *R_f_* was exported into MS Excel, and the respective molecular weights were calculated with the calibration curve drawn by the MW standard. Equation (1) was applied, providing an R2 = 1.00 curve fit.

mw = −8,135,369.29 * *R_f_*^5^ + 26,466,856.04 * *R_f_*^4^ – 33,575,979.43 * *R_f_*^3^ + 20,832,146.87 * *R_f_*^2^ – 6,448,569.1 * *R_f_* + 858,199.79, (1)

### 2.11. WB Panel Test—Presentation of WB-Based Panel Test Results

The interpretation of the PAGE-WB results was carried out by the calculated molecular weights of the recognizable bands on the PAGE-WB lanes, as Figure 3 demonstrates. The analyzed serum samples were divided into three different groups according to the *Brucella* ELISA results: *Brucella* negative, FP, and positive, as Figure 3B–D show. The blue arrow in Figure 3A shows the interpretation process from the WB to the uniform band representation through densitometry with the example of the MW standard. For easier visualization and interpretation, neither the intensity nor the width of the peaks of the densitometry, but only the mw of the bands, was presented. The bands of the serum samples by type are presented as collected lanes as Figures 3 and 4 show, providing a representation of a gel picture.

The consensus mw was calculated for the serum samples according to the CV% of a given group of bands around a certain Rf. The criterion for when a band was included in a consensus mw was ≤2 CV% (far below the acceptance criteria determined for the precision of the method; see the related parameters in the Results, Section 3) based on mw ranges determined by the bands of the MW standard. Consensus was calculated as a percentage of the presence of a certain band in lanes compared to all sample lanes.

### 2.12. Analytical Parameters—Routine Serology Tests

Since our routine laboratory tests are subjects of the annual Interlaboratory Test program provided by the Animal Health Laboratory of the French Agency for Food Environmental & Occupational Health Safety (ANSES) and our results were valid year by year, analytical parameters of routine serology tests were not determined.

### 2.13. Analytical Parameters—WB Panel Test—Precision

Precision parameters of the WB panel test were determined as follows: Retention factors (Rf) for bands from repeated PAGE separations and WBs of the MW standard and the WB panel developed with a *Brucella*-positive reference serum were determined as described above. The Median, Standard Deviation (SD), and Coefficient of Variation (CV%) of the *R_f_*s were calculated according to the experimental setups for repeatability and reproducibility. The acceptance criterion for the precision parameters was generally <5 CV%.

### 2.14. Analytical Parameters—WB Panel Test—Precision—Repeatability

For repeatability, 5 MW STD samples were separated in one PAGE run, and then the gel was Western-Blotted. The MW STD blots were not developed. The lanes underwent densitometry, and from the densitometry results, the CV% of the mw-s of the separated protein bands was calculated.

### 2.15. Analytical Parameters—WB Panel Test—Precision—Reproducibility

For reproducibility, a 1–1 MW STD sample was separated on five different days in different PAGE runs, and then the gels were Western-Blotted. The MW STD blots were not developed. The lanes underwent densitometry, and from the densitometry results, the CV% of the mw-s of the separated protein bands was calculated.

Moreover, the isolated antigen panel was separated on four different days in different PAGE runs, and then the gels were Western-Blotted and developed with CCCP#19 reference serum. The lanes underwent densitometry, and from the densitometry results, the CV% of the mw-s of the separated bands was calculated.

### 2.16. Diagnostic Parameters

Table 2 summarizes how the diagnostic parameters for both the ELISA and the WB-based panel test, that is, Diagnostic Sensitivity (DSe) and Diagnostic Specificity (DSp) [50], and the Positive Predictive Value (PPV) and Negative Predictive Value (NPV) [51], were calculated from the results of the Verified Test (ELISA and WB panel test) and reference results. The reference method was the CFT, which was carried out as the authorized confirmation test according to the WOAH Manual [18], and/or bacteriological examinations and/or diagnostic autopsy with a collective term, the non-serological diagnosis means (NSDs).

## 3. Results

### 3.1. Results of Case Study Conducted with Routine Serological Tests and Swine Sera

**Figure 2:**
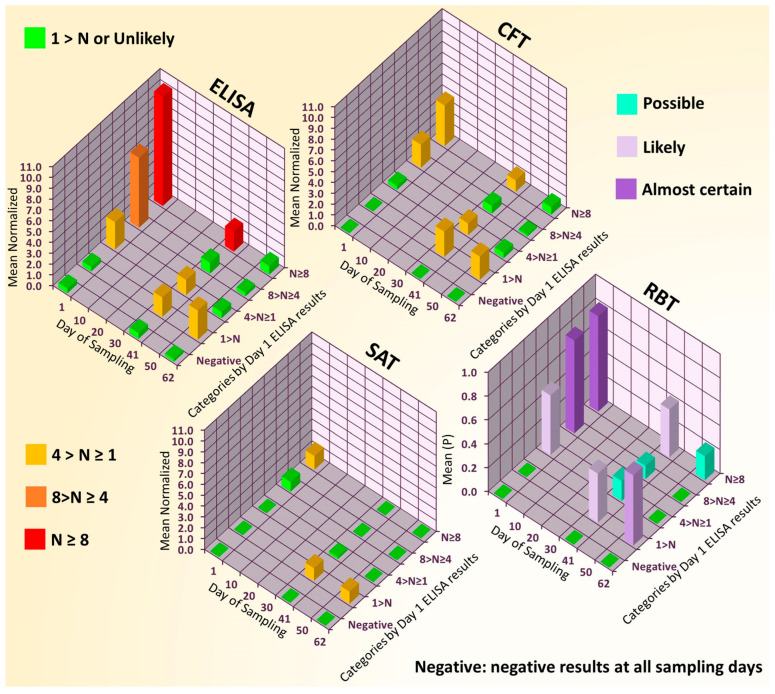
Trends in occurrence of brucellosis seropositivity in data of Hungarian swine livestock (70-member gilt subset) with 62-day follow-up. ELISA, CFT, and SAT results were normalized (to a value N) according to their respective cut-off values (10%, 20 ICFTU/mL, and 30 NE, respectively [17]). The yes/no outcomes of the RBT are represented by 1/0 in the chart, and normalization was not carried out. Individual gilts were categorized according to the normalized ELISA results measured in Day 1 samples—N ≥ 8, 8 > N ≥ 4, 4 > N ≥ 1, 1 > N—and a gilt was classified as negative it showed negative ELISA results at all 3 sampling points, and the means of the categories were calculated. In the case of the RBT, the 0 to 1 scale for the means of the categories (value P) provides a probabilistic interpretation of the positiveness (almost certain: P ≥ 0.75; likely: 0.75 > P ≥ 0.35; possible: 0.35 > P ≥ 0.1; unlikely: 0.1 > P ≥ 0).

A total of 193 serum samples of gilts were obtained for brucellosis screening from a population imported for breeding purposes and considered to be brucellae-free. The analysis of the submitted samples showed extensive brucellosis seropositivity in the herd both with the screening test and the confirmatory tests, as shown in Table 3 and Figure 2. Further sampling and analysis were performed on a subset of the herd (70 specimens for technical/traceability reasons) at two additional points in time (Days 41 and 62).

The herd was eventually condemned to compulsory slaughter, and diagnostic autopsies were carried out on four gilts with tissue sampling for agent-identifying investigations. Bacteriological examinations were performed separately for the available organs (spleen, liver, uterus, and genital lymph nodes), and no bacteria belonging to the genus *Brucella* were detected in the samples either by culturing the bacteria for whole *Brucella* spp., or by the specific molecular diagnostic method (PCR) for the detection of *Brucella suis*. Examination for *Escherichia coli* proved that the persistence of *E. coli* did not differ from the healthy bacteria flora. Intestinal testing for *Salmonella* spp. and a test for *Yersinia enterocolitica* also provided negative results. Negative results are not presented.

### 3.2. Serological Test Results Connected to the WB Panel Test

The results presented in Table 4 mirror the routine operation and the role of our lab as the national reference laboratory. This means that samples that arrived for confirmatory checks from other laboratories were tested by all serologic means (e.g., samples HU 3393424361, HU 3348168414, HU 4156, HU 3073823781/1, HU 3073823781/2, and the latest two samples were sent after resampling), while internal samples with negative ELISA results were not processed further with the other confirmatory serological tests (e.g., sample HU 3214704456), marked in Table 4 as ND. In the case of negative *Brucella* serologic results, other tests were not conducted. Due to the restricted sample volumes, not every serum was tested for *Yersinia*.

### 3.3. WB Panel Test Results

**Figure 3:**
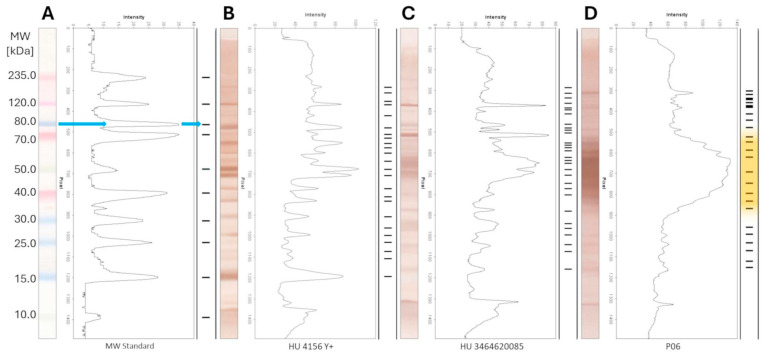
Interpretation of the results of the WB-based panel test. Bands under mw 15 kDa are not shown for serum samples. (**A**) MW STD lane as undeveloped WB; (**B**) *Brucella*-negative, (**C**) False-Positive, and (**D**) *Brucella*-positive lanes as developed WB. Blue arrow: interpretation process. Orange background: unresolvable species.

PAGE gel images before Western Blotting are not shown; only scanned WBs representing samples with different serologic results (*Brucella* negative, positive, and false positive) are shown in Figure 3.

**Figure 4:**
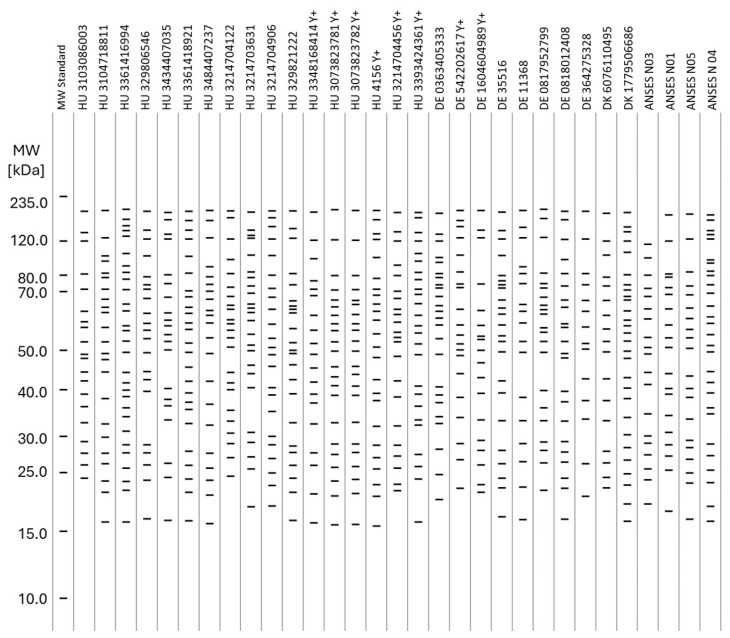
Identified bands on the Western Blots of the negative samples by molecular weight. Bands under 15 kDa mw are not shown for serum samples.

**Figure 5:**
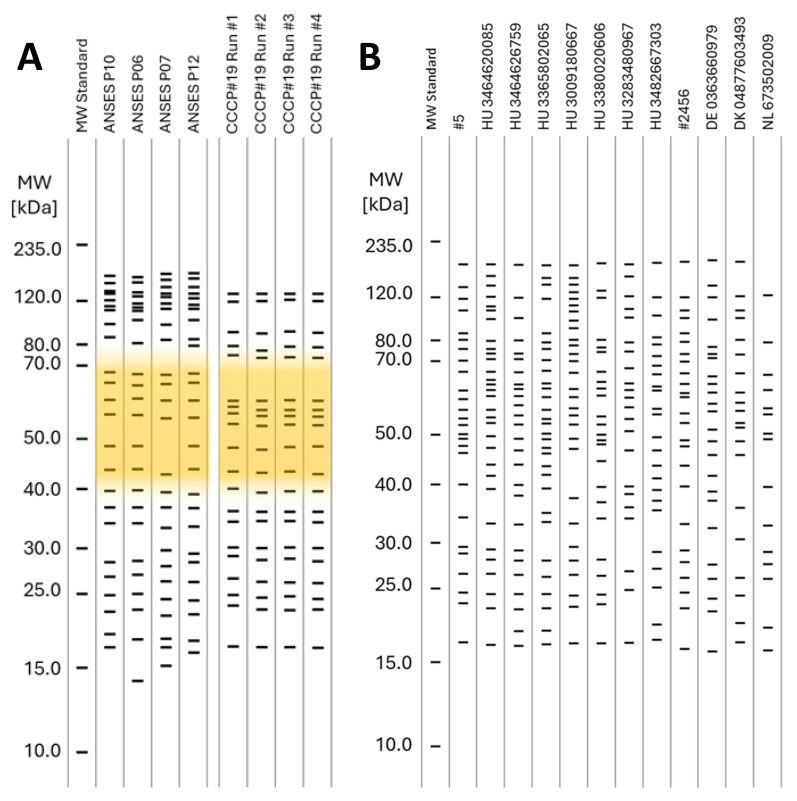
Identified bands on the Western Blots by molecular weight. (**A**): Positive samples. Orange background: unresolvable normally distributed species. (**B**): False-positive samples. Bands under 15 kDa mw are not shown for serum samples.

The serum samples were divided into three groups according to the *Brucella* ELISA results, and the WBs in a certain group are presented accordingly in Figure 3, Figure 4 and Figure 5. The figures present the molecular weight distributions of the isolated *Brucella* proteins (panel) that interacted with antibodies in the serum samples. Representing only the mw provides easier visualization, focusing on only the presence/absence of a certain interaction rather than presenting raw pictures of the WBs or showing the intensity and shape of the densitometry results of a certain band. In the WB of all positive *Brucella* serums, the above-mentioned wide, unresolvable staining with high optical density in the 35.0–75.0 kDa mw range could be identified, as represented with an orange background in Figure 5A. Bands under 15 kDa mw are not shown for serum samples since these sizes are considered protein fragments only. A sharp difference can be observed in the range of the high-molecular-weight proteins (above 100.0 kDa) in the case of positive sera, as the marked range is densely populated in the ANSES samples and only two bands were detected in the CCCP#19 control serum. This difference can be attributed to the extremely long storage time (35 years between purchase and processing) of the CCCP#19 serum, causing the degradation of proteins with a high mw.

**Figure 6:**
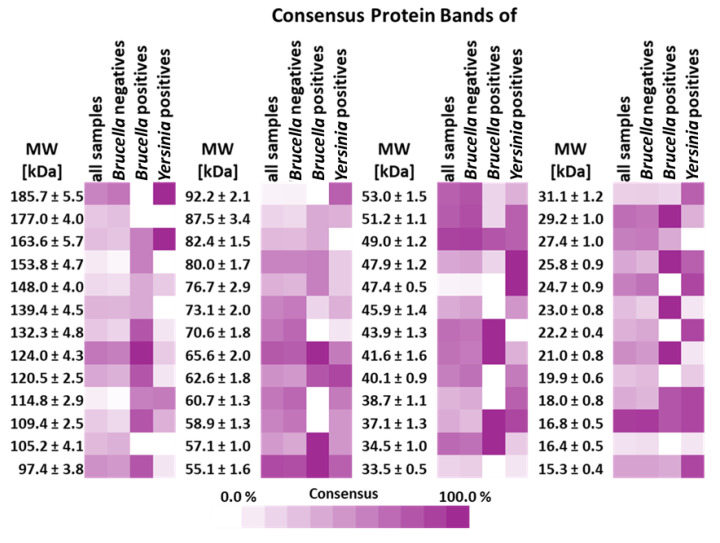
Heat map of the consensus protein bands identified on the Western Blots of all sample runs by molecular weight. Consensus mw was calculated for the serum samples according to the CV% of a given group of bands around a certain *R_f_*. The criterion for when a band was included in a consensus mw was ≤2 CV% based on mw ranges determined by the bands of the MW standard. Consensus was calculated as a percentage of the presence of a certain band in lanes compared to all sample lanes. Bands under 15 kDa mw are not shown for serum samples.

Heat maps of the consensus protein bands were established according to the consensus described above for the WBs of the sample runs and are presented in Figure 6 to identify unambiguous differentiating patterns/protein panels. However, as mentioned above, there were neither identifiable individual protein bands nor a panel of bands with a high consensus score in the positive *Brucella* sera differentiating the positive and negative serums unambiguously.

### 3.4. Analytical Parameters of WB Panel Test

Table 5 and Table 6 show the determined repeatability and reproducibility precision parameters. Although the accuracy of the PAGE separation defined by the MW STD was not within the scope of this study, Table 6 demonstrates that the difference between the nominal molecular weights and the determined ones never exceeds 7.0% for the whole separation range. Both repeatability and reproducibility remain under the acceptance criterion for the whole range, with means of 1.36 CV% for both parameters.

Reproducibility determined with a positive serum sample (CCCP#19) also remains under the acceptance criterion for the whole range, never exceeding 2 CV%, with a mean of 0.70 CV%, as seen in Table 6.

### 3.5. Diagnostic Parameters of Routine Brucella ELISA and WB Panel Test

The CFT and/or the non-serological diagnostic means represented the reference method for the determination of the diagnostic parameters of both the routine *Brucella* ELISA and the WB panel test. However, as shown in Table 4, in some negative cases, the CFT was not carried out, and the NSDs were applied only to animals that displayed a positive result. Thus, reference results were considered negative if neither a CFT nor NSD result was available and the ELISA resulted in negative diagnosis. This approach was applied in the determination of the diagnostic parameters presented in Table 7 and Table 8.

As shown in the heat map presented in Figure 6 and as will be discussed later, there were no identifiable individual protein bands differentiating the positive and negative serums unambiguously, and even considering a panel of bands with a high consensus score in the positive *Brucella* sera, unambiguous differentiation was unlikely. However, one characteristic feature in the WB of positive *Brucella* sera could be identified: the wide, unresolvable staining with high optical density in the 35.0–75.0 kDa mw range, represented with the orange background in Figure 3D and Figure 5. The presence of such a feature was considered as an unambiguous marker of *Brucella* positivity, and conversely, the absence of it proved negativity. This approach was applied to fill up the contingency table, to determine the diagnostic parameters of the Western Blot panel test, and to mark positivity/negativity in the respective columns of Table 4.

## 4. Discussion

### 4.1. A Case Study Conducted with Routine Serological Tests and Swine Sera

The analysis of the submitted samples showed extensive brucellosis seropositivity in the herd using both the screening test and the confirmatory tests, as shown in Table 3 and Figure 2. Bacteriological examinations of tissue samples from diagnostic autopsies proved the absence of bacteria belonging to the genus *Brucella* and the persistence of *E. coli* identical to the healthy bacteria flora. Moreover, the absence of both *Salmonella* spp. and *Yersinia enterocolitica* was also confirmed.

The main conclusions of this case study are as follows: (i) the confirmation tests may actually confirm the false-positive results provided by the ELISA test—it is mainly their substantially lower analytical sensitivity that proves the opposite in some cases; (ii) there are more cross-reacting Gram-negative bacterium species than we generally consider to contribute to false positives [19], and an infection caused by an unidentified agent has swept over the whole population, provoking a transient immune reaction only; and finally, (iii) the commonality in both the screening ELISA and the confirmatory tests causing false positivity is the antigen applied.

### 4.2. Relevance of the Case Study Results in the Context of the WB Panel Test

Although the case study was conducted with swine sera and the WB panel test was developed with and targeted bovine serums, the conclusions of the two approaches can be merged since (i) the origin of the antigen is the same *Brucella abortus* strain 99 (Weybridge) (S99)3 for both swine and bovine tests according to WOAH Chapter 3.1.4.B.2.2. [18], and consequently, (ii) the occurrence of false-positive serology results is similar.

### 4.3. Limitations of the WB Panel Test

Before discussing the results, we should disclose the limitations of the study. The main limitation stems from the success story of the official *Brucella* infection status of European bovine livestock mentioned in the Introduction. Consequently, positive sera of naturally infected animals are theoretically unavailable in the EU. In this case, theory coincides with practice: due to the *Brucella*-free status of the EU member states, it is almost impossible to acquire such positive samples even from outside of the Union for administrative reasons. This constraint limited the positive samples used to two: (i) the official ANSES positive standard set and (ii) one very old sample from the former Soviet Union. Consequently, the statistical power of the study derived from this limited number of positive serums is weak, and certainly, the representativeness of the results is also limited.

A further limitation of the application of the WB technique is the resolution of the scanned images, that is, the maximum of 600 dpi provided by the flatbed scanner used. A higher scanning resolution and more precise densitometry of advanced gel documentation systems (both hardware and software) can give higher precision. With such a system, the more sensitive, fluorescence-based staining/development of WBs can also be accomplished, providing even higher precision.

### 4.4. Interpretation of the Results of the WB Panel Test

The results of this study provided additional confirmation that false-positive results still haunt brucellosis serology. The serology of the livestock serum specimens measured as part of the WB panel test study resulted in 12 false positives out of 39 samples. The confirmation serologic tests carried out with these FPs resulted in similarly confusing outcomes (see Table 4), as presented in the case study (see Table 3 and Figure 2) in the Introduction, which was pointed out previously in the literature [11,12].

Even though our efforts to isolate and analyze dozens of different *Brucella* proteins against positive and negative sera in the same run (WB panel) was successful, as Figure 6 shows, even measuring a wide panel of antigens cannot solve the FPSR problem unambiguously at the protein level. The set of protein bands with approximate molecular weights of 65, 57, 43, 41, 34, 23, and 21 kDa identified with 100% detectability by *Brucella*-positive serums only provides uncertain differentiation, since the estimated presence of a certain band in the negative sera is also 76, 42, 65, 63, 67, 23, and 49%, respectively. If each protein is considered individually, the situation is even more dire. The less overlapping individual protein with 47.4 kDa that shows 7% presence in the negatives proves to be unrecognizable by the *Brucella*-positive serum (0%). If the 153.8 kDa band with 4.7% presence in the negatives is chosen, only 60% presence in the *Brucella* positives is obtained, or, in the case of the 114.8 kDa band, 60% presence in both the *Brucella* and *Yersinia* positives is detected, proving that the antigenicity of the *Brucella* vs. *Yersinia* proteins is still indistinguishable.

Despite the unsatisfactory results with discrete protein bands, a resolution for the FPSR problem was nevertheless found. This solution utilizes the exclusive properties of Western Blotting to detect specific antigen–antibody interactions formed not only at the protein level but also in the higher organizational structure of the bacteria. During the analysis of the WB of positive *Brucella* sera, wide, unresolvable staining with high optical density in the 35.0–75.0 kDa range with a normal-like molecular weight distribution was identified, as presented in the densitometry section of Figure 3D (represented thereafter with an orange background in the figures related to positive results). The presence of such staining was considered an unambiguous marker of *Brucella* positivity, and conversely, the absence of it proved negativity in the framework of the study (see Limitations).

The nature of this unresolvable staining is quite ghostlike, as it is detectable only after the adequate separation of *Brucella* antigens that are to be individually targeted by the serum antibodies. Consequently, the presence of the molecular species represented by the staining can be detected by the applied method only, while their origin remained in twilight.

Although this is not within the scope of this study, we can speculate to dispel the gloom around the origin of the unresolvable staining. First, both the culturing of the bacteria (killing the cells with peracetic acid) and the lysis of the cells (by TFA) focus on maintaining the protein’s natural status as much as possible. However, both the peracetic acid and the TFA cause the acidic hydrolysis of the periplasmic peptidoglycan polymers [52] in an uncontrolled manner, theoretically generating poly- and oligomers with chain lengths normally distributed from 1 kDa to 25 kDa. Second, several proteins from the outer cell membranes (mostly outer membrane proteins, OMPs [34,53]) are covalently bound to the amino acid terminal of the peptidoglycan monomers [54], providing connections between the normally distributed peptidoglycan species and the separated and immunostained proteins in the WBs. Third, the principles of SDS PAGE provide the separation of uniformly charged and shaped molecules by their molecular weight; thus, the presence of unresolvable stains suggests the presence of poly- or oligomeric, non-protein derivatives covalently attached to the proteins—exactly like the residues of the hydrolyzed peptidoglycan layer. Seemingly, the peptidoglycan derivatization of the OMP can explain the presence of the unresolved immunostaining, but why this feature characterizes only the *Brucella*-positive serums remains obscure.

Considering either animal husbandry conditions (related to the breeding regions (see Table 2)) or possible *Yersinia* infections (see Table 5), identifiable differences in the WB patterns of the negative and false-positive sera were not found, which suggests the same microbial exposure in the wide geographic area in Europe. All these results demonstrate (i) the presence of numerous (unidentified) bacteria species (most probably Gram-negative) provoking similar immune responses in the hosts, and consequently, (ii) an extremely high level of homology among the proteins of the Gram-negative bacteria determining their antigenicity.

The uniformity of the non-*Brucella* serums, which strikingly differ from the *Brucella*-positive sera in the WB results, suggests that the origin of this difference could be the intracellular lifestyle of *Brucella* [5,6,55], resulting in an entirely different immune response of the host, which is mirrored by the antibody composition of the serums.

## 5. Conclusions

Ghostbuster, a Western Blot-based panel method for resolving false-positive serology results in the diagnosis of brucellosis, was developed. Within limits, the method discriminates *Brucella*-positive sera from samples which have false positivity related to infections caused by other Gram-negative bacteria, with better diagnostic parameters compared to routine serologic tests.

The sample throughput of the Ghostbuster method is not high enough for it to be used for screening; however, it represents a relatively cost-effective, easily standardizable, and rapid method to be used in parallel with existing confirmation methods. The development of the pre-prepared WB membranes provides a maximum 3 h analysis time and requires no special skills and equipment, and the shelf life of the membranes is adequate for laboratory validation. In appropriate laboratories, cell cultivation and bacterial protein isolation for protein antigen isolation are relatively simple and straightforward. Both SDS PAGE separation and Western Blotting (including development and densitometry) can be accomplished with commercially available equipment, ready-to-use reagents, and open-source software. Naturally, the application of advanced gel documentation hardware and software systems can provide even higher precision than that which we reached in this study.

Due to the above-mentioned limitations, however, repetition of the measurement with an adequate number of positive serum samples from naturally infected animals is required to confirm our results. As this can only be accomplished in laboratories outside of the EU and has not been attained and is not attainable by the authors, we encourage researchers interested in this confirmation to repeat our work with a broader range of natural samples, complemented with dilution experiments accomplished using positive and negative samples drawn from animals kept in similar circumstances.

Nevertheless, the results of this study could attract broader attention, since the non-protein molecular species in the 35.0–75.0 kDa range after proper identification could also contribute to the application of OMPs as antigens in *Brucella* serology, and the same fraction can be considered in the development of synthetic *Brucella* vaccines. Nota bene, WB membranes are ideal for both Edman and mass spectrometry peptide sequencing.

## Figures and Tables

**Table 1:** Bovine serum samples analyzed. HU: animal with Hungarian ID; DE: German ID; DK: Danish ID; and NL: Dutch ID. a: duplicated sample.

Hungarian IDs		Reference Samples	Imported IDs
HU 3214704456	HU 3484407237	CCCP#19	DE 0363405333
HU 3393424361	HU 3214704122	ANSES BRU POS SR06	DE 35516
HU 3348168414	HU 3214703631	ANSES BRU POS SR07	DE 11368
Need no explanation HU 4156	HU 3214704906	ANSES BRU POS SR10	DE 0817952799
HU 3073823781/1 ^a^	HU 329821222	ANSES BRU POS SR12	DE 0818012408
HU 3073823781/2 ^a^	HU 3464620085	ANSES BRU NEG SR01	DE 364275328
HU 3103086003	HU 3464626759	ANSES BRU NEG SR03	DE 542202617
HU 3104718811	HU 3365802065	ANSES BRU NEG SR04	DE 1604604989
HU 3361416994	HU 3009180667	ANSES BRU NEG SR05	DE 0363660979
HU 329806546	HU 3380020606	–	DK 6076110495
HU 3434407035	HU 3283480967	–	DK 1779506686
HU 3361418921	#2456	–	DK 04877603493
HU 3482667303	#5	–	NL 673502009

**Table 2:** Contingency table for calculation of diagnostic parameters. T—Number of Samples; T = TP + TN + FP + FN; P—Positive; N—Negative; TP—True Positive; TN—True Negative; FP—False Positive; FN—False Negative; PP—Predicted Positive; PN—Predicted Negative; PPV—Positive Predictive Value; NPV—Negative Predictive Value; DSe—Diagnostic Sensitivity; DSp—Diagnostic Specificity.

		Reference Method			
	Result	Positive	Negative	Total	
**Verified Test**	**Positive**	TP	FP	PP = TP + FP	PPV = TP/PP
	**Negative**	FN	TN	PN = TN + FN	NPV = TN/PN
	**Total**	P = TP + FN	N = TN + FP	T	
		DSe = TP/P	DSp = TN/N		

**Table 3:** Time courses of the positivity of test results [%] by tests performed for the whole herd (initial sampling, *n* = 193) and for the resampled subset (*n* = 70).

Positivity of Test Results [%]				
Test	Whole Herd (*n* = 193)	Resampled Population (*n* = 70)		
		Day of Sampling		
		1st	41st	62nd
**ELISA**	51.8	41.4	27.1	24.3
**CFT**	13.7	31.4	32.6	24.3
**SAT**	7.4	15.7	4.3	14.3
**RBT**	13.1	30.0	17.1	15.7

**Table 4:** Test results. Column order is set according to the test type (screening (SCR), confirmation (CON), or investigation (INV)) and sample type (serum or other, like bacteriology (BCT), PCR, or diagnostic autopsy (DAP)). Serum tests: ELISA, CFT, SAT, RBT for *Brucella*, and serum ELISA test for *Yersinia* (YER). WB panel test: WBP. Positive or negative results are shown, only represented with (+) or (−), respectively, without raw data. Where positivity was determined by other means, the + was substituted with the analysis type. N/A: not applicable; ND: not determined.

**Sample ID** **(ELISA Negatives,** **Hungarian)**	**HU 3214704456**	**HU 3393424361**	**HU 3348168414**	**HU 4156**	**HU 3073823781/1**	**HU 3073823781/2**	**HU 3103086003**	**HU 3104718811**	**HU 3361416994**	**HU 329806546**	**HU 3434407035**	**HU 3361418921**	**HU 3484407237**	**HU 3214704122**	**HU 3214703631**	**HU 3214704906**	**HU 329821222**
**ELISA/SER/SCR**	(−)	(−)	(−)	(−)	(−)	(−)	(−)	(−)	(−)	(−)	(−)	(−)	(−)	(−)	(−)	(−)	(−)
**CFT/SER/CON**	ND	(−)	(−)	(−)	(−)	(−)	ND	ND	ND	ND	ND	ND	ND	ND	ND	ND	ND
**SAT/SER/CON**	ND	(−)	(−)	(−)	(−)	(−)	ND	ND	ND	ND	ND	ND	ND	ND	ND	ND	ND
**RBT/SER/CON**	ND	(−)	(−)	(−)	(−)	(−)	ND	ND	ND	ND	ND	ND	ND	ND	ND	ND	ND
**OTR/OTR/CON**	ND	ND	ND	ND	ND	ND	ND	ND	ND	ND	ND	ND	ND	ND	ND	ND	ND
**YER/SER/INV**	(+)	(+)	(+)	(+)	(+)	(−)	ND	ND	ND	ND	ND	ND	ND	(−)	(−)	(−)	(−)
**WBP/OTR/INV**	(−)	(−)	(−)	(−)	(−)	(−)	(−)	(−)	(−)	(−)	(−)	(−)	(−)	(−)	(−)	(−)	(−)
**Sample ID** **(ELISA Negatives,** **Other EU)**	**DE 0363405333**	**DE 35516**	**DE 11368**	**DE 0817952799**	**DE 0818012408**	**DE 364275328**	**DE 542202617**	**DE 1604604989**	**DK 6076110495**	**DK 1779506686**	**ANSES BRU NEG SR01**	**ANSES BRU NEG SR03**	**ANSES BRU NEG SR04**	**ANSES BRU NEG SR05**			
**ELISA/SER/SCR**	(−)	(−)	(−)	(−)	(−)	(−)	(−)	(−)	(−)	(−)	(−)	(−)	(−)	(−)			
**CFT/SER/CON**	ND	ND	ND	ND	ND	ND	ND	ND	ND	ND	(−)	(−)	(−)	(−)			
**SAT/SER/CON**	ND	ND	ND	ND	ND	ND	ND	ND	ND	ND	(−)	(−)	(−)	(−)			
**RBT/SER/CON**	ND	ND	ND	ND	ND	(−)	(−)	(−)	ND	ND	(−)	(−)	(−)	(−)			
**OTR/OTR/CON**	ND	ND	ND	ND	ND	(−)	(−)	(−)	ND	ND	N/A	N/A	N/A	N/A			
**YER/SER/INV**	ND	ND	ND	ND	ND	(−)	(+)	(+)	ND	ND	ND	ND	ND	ND			
**WBP/OTR/INV**	(−)	(−)	(−)	(−)	(−)	(−)	(−)	(−)	(−)	(−)	(−)	(−)	(−)	(−)			
**Sample ID** **(ELISA Positives)**	**HU 3482667303**	**HU 3464620085**	**HU 3464626759**	**HU 3365802065**	**HU 3009180667**	**HU 3380020606**	**HU 3283480967**	**#2456**	**#5**	**NL 673502009**	**DK 04877603493**	**DE 0363660979**	**CCCP#19**	**ANSES BRU POS SR06**	**ANSES BRU POS SR07**	**ANSES BRU POS SR10**	**ANSES BRU POS SR12**
**ELISA/SER/SCR**	(+)	(+)	(+)	(+)	(+)	(+)	(+)	(+)	(+)	(+)	(+)	(+)	(+)	(+)	(+)	(+)	(+)
**CFT/SER/CON**	(−)	(+)	(+)	(+)	(−)	(+)	(+)	(−)	(−)	(+)	ND	ND	(+)	(+)	(+)	(+)	(+)
**SAT/SER/CON**	(−)	(+)	(+)	(+)	(+)	(+)	(−)	(−)	(+)	(+)	(+)	(+)	(+)	(+)	(+)	(+)	(+)
**RBT/SER/CON**	(−)	(−)	(+)	(+)	(+)	(+)	(+)	(−)	(+)	(+)	(+)	(+)	(+)	(+)	(+)	(+)	(+)
**OTR/OTR/CON**	ND	(−)	(−)	(−)	(−)	(−)	(−)	(−)	(−)	(−)	(−)	(−)	N/A	N/A	N/A	N/A	N/A
**YER/SER/INV**	(−)	ND	ND	(−)	(+)	(+)	(−)	(+)	(+)	(+)	(−)	(−)	ND	ND	ND	ND	ND
**WBP/OTR/INV**	(−)	(−)	(−)	(−)	(−)	(−)	(−)	(−)	(−)	(−)	(−)	(−)	(+)	(+)	(+)	(+)	(+)

**Table 5:** Precision parameters in CV% determined for the WB panel test defined on the MW STD bands. The nominal mw was provided by the manufacturer.

	Precision Parameter			
	Repeatability		Reproducibility	
Nominal mw	mw [kDa]	CV [%]	mw [kDa]	CV [%]
**235.0 kDa**	234.8	0.7	230.0	1.7
**120.0 kDa**	120.1	0.5	120.2	1.2
**80.0 kDa**	81.9	0.2	82.2	0.6
**70.0 kDa**	70.4	0.8	70.9	0.6
**50.0 kDa**	50.7	0.6	51.1	1.2
**40.0 kDa**	42.1	1.3	42.1	1.0
**30.0 kDa**	32.0	1.3	32.0	0.9
**25.0 kDa**	24.2	1.9	24.3	1.5
**15.0 kDa**	15.9	0.9	15.9	1.0
**10.0 kDa**	10.2	5.0	10.2	3.1
**Mean**	**–**	**1.3**	**–**	**1.3**

**Table 6:** Reproducibility in CV% determined for the WB panel test defined on bands detected during the four runs carried out with the CCCP #19 positive standard serum.

Reproducibility					
mw [kDa]	CV [%]	mw [kDa]	CV [%]	mw [kDa]	CV [%]
129.5	0.00	53.6	0.57	30.8	0.22
118.9	0.89	51.8	0.31	28.8	1.34
87.8	0.73	49.0	0.92	25.0	1.30
78,1	1.52	47.9	0.50	22.8	1.07
72,4	0.87	43.8	0.47	21.1	1.31
64,9	1.12	40.7	0.68	17.1	0.25
57.0	0.29	37.1	0.15	–	–
54.8	0.67	35.4	0.31	–	–
**Mean**					**1.3**

**Table 7:** Contingency table and the determined diagnostic parameters for the sample set measured by ELISA. Reference results were provided by the CFT and/or non-serological diagnostic means.

		Reference Method			
	Result	Positive	Negative	Total	
**ELISA**	**Positive**	5	12	PP = 17	PPV = 0.29
	**Negative**	0	31	PN = 31	NPV = 1.00
	**Total**	P = 5	P = 43	48	
		DSe = 1.00	DSp = 0.73		

**Table 8:** Contingency table and the determined diagnostic parameters for the sample set analyzed with the WB panel test. Reference results were provided by the CFT and/or non-serological diagnostic means.

		Reference Method			
	Result	Positive	Negative	Total	
**WB PT**	**Positive**	5	0	PP = 5	PPV = 1.00
	**Negative**	0	43	PN = 43	NPV = 1.00
	**Total**	P = 5	N = 43	48	
		DSe = 1.00	DSp = 1.00		

## Data Availability

The original contributions presented in this study are included in the article. Further inquiries can be directed to the corresponding authors.
